# The role of *Nucularia perrinii* Batt. (Chenopodiaceae) in the camel-based Sahrawi social-ecological system

**DOI:** 10.1186/s13002-017-0141-3

**Published:** 2017-02-08

**Authors:** Gabriele Volpato, Antonello Di Nardo

**Affiliations:** 1 Center for Integrative Conservation Research, University of Georgia, Athens, GA USA; 2 The Pirbright Institute, Pirbright, Woking, Surrey, UK

**Keywords:** Pastoral nomads, Salty pastures, Dromedary camel, Sahara, Grazing resources, Milk taste, Cultural identity

## Abstract

**Background:**

Pastoral social-ecological systems (SESs) are adaptive and complex systems rooted in the extensive exploitation of forage plants for livestock-based livelihoods and culture. There are species and relations that are foundational to the existence of these SESs. *Nucularia perrinii* Batt. (Chenopodiaceae) is an endemic halophyte plant of central and western Sahara seldom cited in the scientific literature. The objective of this study was to investigate the role of this plant in the SES of the Sahrawi camel nomads of Western Sahara.

**Methods:**

The data analyzed were collected in the Sahrawi refugee camps of Algeria and in Western Sahara between 2006 and 2010. Fieldwork included semi-structured (*n* = 38) and retrospective (*n* = 12) interviews with Sahrawi refugees, nomads, and camel owners about *N. perrinii* and associated topics (e.g. distribution, importance for camels, camel diseases, associated grazing practices, other forage plants, etc.).

**Results:**

*Askaf*, as the Sahrawi call the plant, is crucial to camels’ survival, providing salts and water even during dry spells. It holds a pivotal role in the Sahrawi culture, defining the geographical boundaries of the Sahrawi SES and relating the grazing territory with the taste it gives to camel milk, which support the inclusion of *askaf* as a main element of Sahrawi cultural identity.

**Conclusions:**

We argue that *N. perrinii* ties the ecology of the western Sahara desert with camel husbandry and associated livelihoods, and further with the culture and worldview of the Sahrawi nomads. We stress the keystone role that some forage plants may have in extensive pastoral SESs worldwide.

## Background

‘Social-ecological systems’ (SESs) are defined as complex, integrated systems in which humans and nature co-evolve, emphasising, with the use of this term, the artificial and arbitrary delineation between a social and an ecological realm [1, 2]. The SES approach recognises that there is an intimate interaction between local ecosystems and their dynamics, on the one hand, and the social, cultural, and economic characteristics and dynamics of communities and societies, on the other hand. SESs are based on specific sets of animal and plant species (including humans) and on their interactions [3, 4]. Pastoral SESs, in particular, depend on livestock species, on forage plants, on the technical relation deployed to feed livestock (e.g. mobility), and on the social and cultural norms and beliefs that glue these relations in a whole [5]. Grazing species, livestock portfolio, and humans are the backbone of complex systems aimed at utilising marginal territories through technical relations based on humans’ and livestock mobility [6, 7]. Among the forage species, one or more of them may have a key role in providing subsistence to distinct livestock species. These plants have deep material and cultural importance among distinct pastoral populations: they define and influence pastoralists’ grazing territories and livelihood practices; they are objects of knowledge accumulation and sharing (e.g. naming and terminology, ethnobiological and ethnoecological knowledge); they have important roles in narratives or symbolism (e.g. the species is tied to myths and ancestors); and they have a unique position in cultural identity (i.e. in defining human groups and their territories, beliefs, and values).

**Fig. 1:**
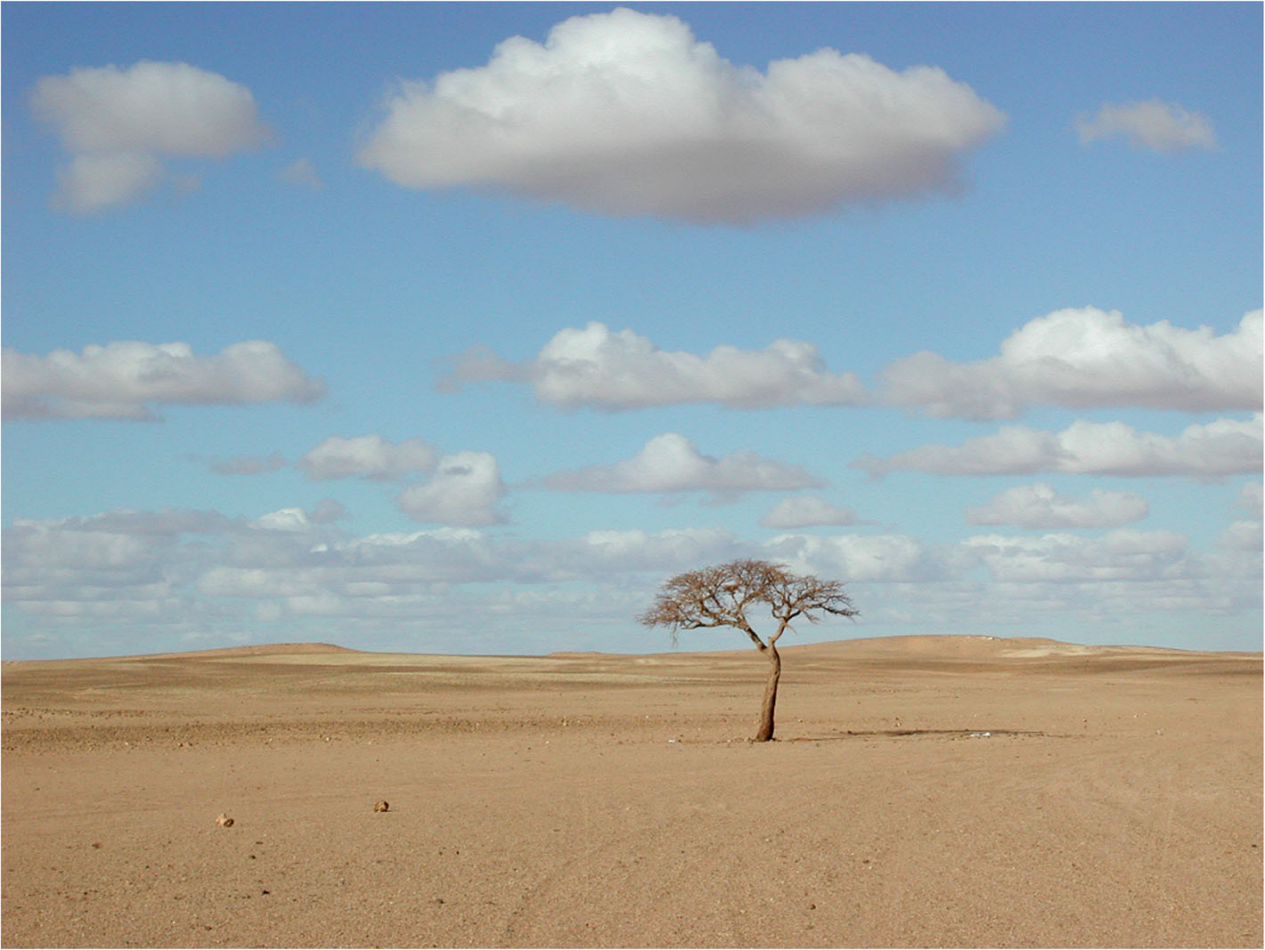
A solitary *Acacia* tree in the barren landscape of the Hammada of Tindouf (GV)

**Fig. 2:**
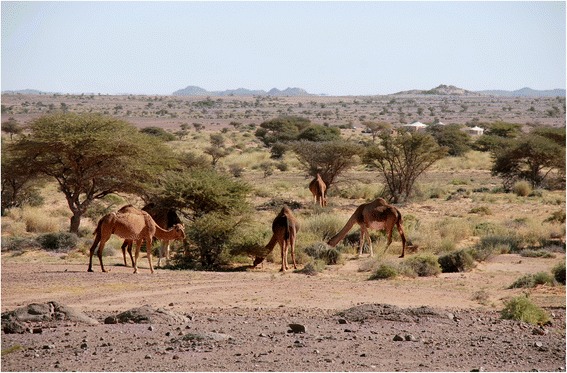
The Acacia-Panicum vegetation of Zemmur (GV)

**Fig. 3:**
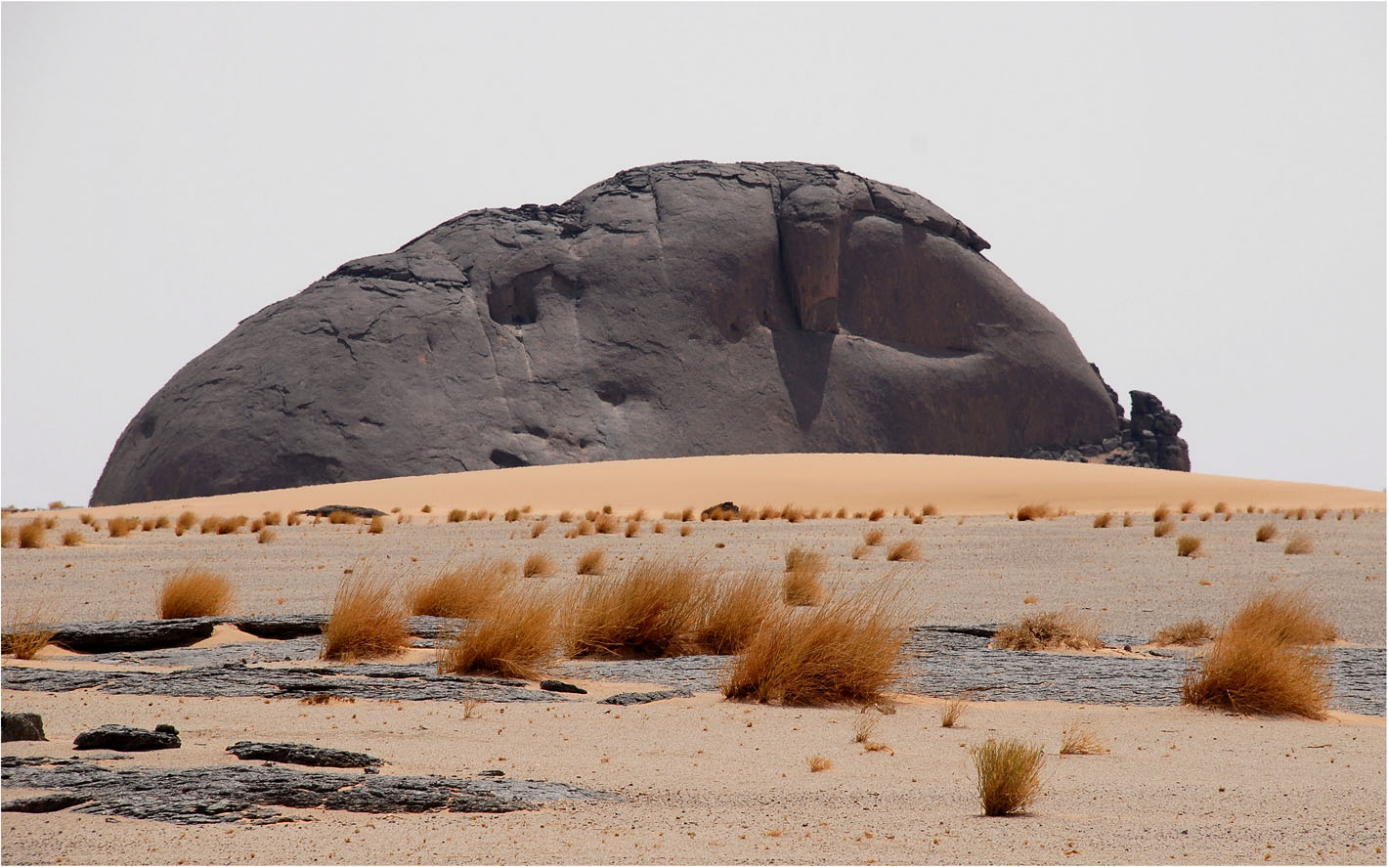
A black granite hill in Tiris (GV)

To explore these themes, we address the material and cultural importance of grazing species in extensive pastoral SESs focusing on the role of *Nucularia perrinii* Batt. (Chenopodiaceae) in the SES of the Sahrawi camel nomads of Western Sahara. The Sahrawi have relied on camels (as well as on small ruminants), on local desert resources, and on a high degree of mobility for their livelihoods for about fifteen hundred years. They traditionally inhabited coastal areas of northwestern Africa including Western Sahara, Northern Mauritania, and part of Southwestern Algeria, and were socially organised into different tribes (e.g. Reguibat, Oulad Delim, Oulad Tidrarin, etc.) [8–11]. In 1975, following Morocco’s occupation of Western Sahara, about 70,000 Sahrawi fled the Moroccan army [12], becoming refugees. Nowadays, after sixteen years of war (1975–1991) between Morocco and the Sahrawi’s armed political organization, the Polisario Front, and the exclusion of refugees from most of their former grazing territories, about 165,000 Sahrawi live in four refugee camps located on a desert plateau called Hamada, close to the Algerian town of Tindouf [13, 14]. In the camps, refugees rely on food aid [12], while seeking to improve their quality of life through an informal economy, engaging in remunerated labor (e.g. as butchers, mechanics, construction workers, etc.) and expanding trading routes through the camps from Mali, Mauritania, Algeria, and Spain [13, 15]. Besides the camps, the Sahrawi have political control over the Eastern part of the Western Sahara, the so-called ‘liberated territories’. Pastoral areas within the ‘liberated territories’ and their biological resources are important to the refugees’ struggle to recover traditional livelihoods and cultural and social practices, from camel husbandry to medicinal plant use [16, 17], as well as to engage in income generating activities [18]. By accessing and using these lands, refugees reduce their dependence on food aid by recovering a much-desired traditional lifestyle and authentic food products, with their taste, and associated cultural values [14, 15, 19]. Today, camel husbandry is practiced in the ‘liberated territories’ (within the regions of Zemmur to the north and Tiris to the south; Figs. 1, 2, and 3) as well as, to a lesser extent, in the surroundings of the refugee camps. Across these areas, the climate is arid and continental, [20], and average annual rainfall is of 30–50 mm,, with recurrent droughts. The most important pastures are *Acacia tortilis* Hayne, *N. perrinii*, *Salsola tetrandra* Forssk., *Panicum turgidum* Forssk., and *Astragalus* and *Stipagrostis* species [21]. The presence of these pastures, coupled with the adaptation of camels to dry environments, make human utilisation of the desert possible. Among the more than one hundred plant species characterising the grazing resources of western Sahara^1^, a central role and position is given to an endemic halophyte plant, *N. perrinii* or *askaf*, as it is locally called.

In spite of its importance for pastoralism in western Sahara, there is little information in the literature about this small desert shrub; there are no studies about its ecology or its chemical content, and it is usually cited only in ethnographic accounts about western Saharan nomads and in regional plant lists [10, 22–27]. Typing ‘Nucularia perrinii’ into Web of Science (http://webofknowledge.com/UA) returns just 1 hit, from our own previous work on Sahrawi pastoralism [28], whereas typing ‘Nucularia perrini’ (the form in which the scientific name is often misspelled) results in 2 hits, related to research on desert locusts [29, 30]. In Google Scholar (https://scholar.google.com/), the figures are of 24 and 63 hits, respectively. Besides reports of its endemic condition in western and central Sahara, few information are available about its real distribution [31]. Bromatological studies have been recently conducted by Correra [32].

In this paper, we discuss the importance of *N. perrinii* for the Sahrawi camel-based SES and for contemporary Sahrawi refugees in the camps of west Algeria. We first describe the methodology used for this study. Thus, we present and discuss the results focusing first on the botanical (e.g. ecology, distribution), ethnobotanical (e.g. direct uses), and ethnotaxonomic aspects of *N. perrinii*, and then on its key role for camel health and survival in western Sahara and on the strategies adopted by camel nomads to lead their camels to graze from *askaf*. We further discuss its cultural importance in terms of the taste that it gives to camel milk, in terms of its role in defining the Sahrawi’s customary territory and its favourable characteristics for camel husbandry, and, based on all this, on its role to define Sahrawi cultural and political identity.

## Methods

The data analysed in this paper were collected in the Sahrawi refugee camps and in the Polisario-controlled ‘liberated territories’ of Western Sahara between 2006 and 2010. Ethnobotanical and ethnobiological fieldwork was carried out in accordance with standard texts [33–35], consisting of anthropological fieldwork methods such as participant observation and interviews [36, 37]. Fieldwork included semi-structured (*n* = 38) and retrospective (*n* = 12) interviews with Sahrawi refugees, nomads, and camel owners about *N. perrinii* and associated topics (e.g. distribution, importance for camels, camel diseases, associated grazing practices, other forage plants, etc.). Interviews were conducted in Hassaniya (the Arabic language with a Berber substrate spoken by the Sahrawi) and Spanish: a local research assistant asked the questions in Hassaniya and translated the answers back into Spanish, which is the second most frequently spoken language among the Sahrawi. To ensure that, during the interview process, no mistakes were made in translation and to clarify doubtful information, interviews were recorded and transcribed with the help of the same research assistant. Qualitative data were coded and analyzed narratively (description, explanation, interpretation, quotations) using NVivo 9 (QSR International Pty Ltd.).

Botanical observations on the vegetation of the liberated territories and on the presence and distribution of *N. perrinii* were conducted and merged with data retrieved from the literature about the species’ distribution. Voucher specimens were collected with informants through a ‘walk in the woods’ approach [34] in the Hamada of Tindouf and across the ‘liberated territories’, on five independent missions between 2006 and 2009. Plant nomenclature follows the Sahara and Western Sahara botanical standard treatises [26, 27, 38, 39] and the International Plant Name Index (www.ipni.org). Voucher specimens were deposited in the National Herbarium of The Netherlands (Wageningen Branch – Herbarium Vadense). Herbarium specimens for *N. perrinii* are GV1047 and GV2042.

## Results and discussion

### Botanical and phytogeographical aspects

**Fig. 4:**
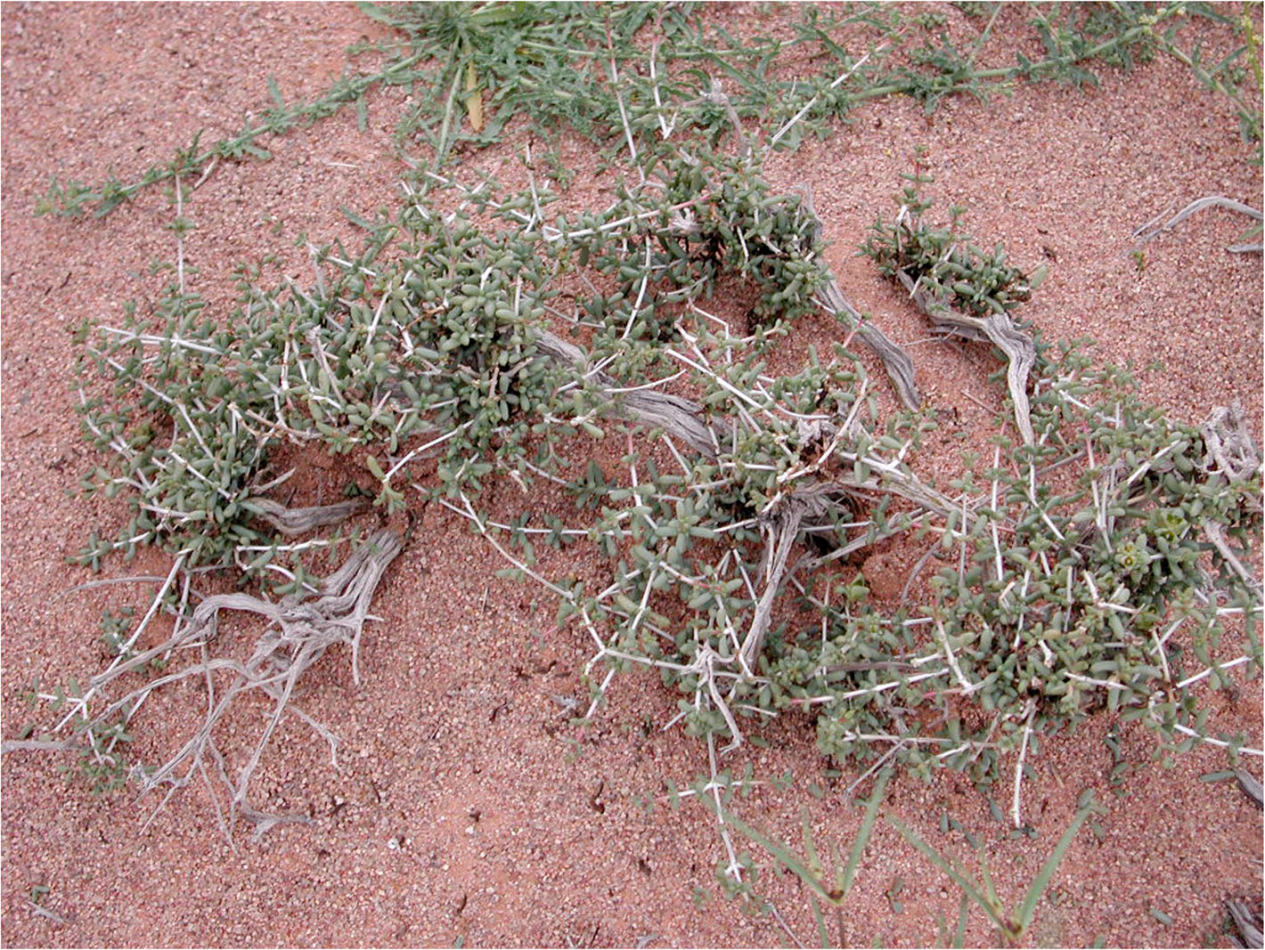
Plant of *N. perrinii* (GV)

**Fig. 5:**
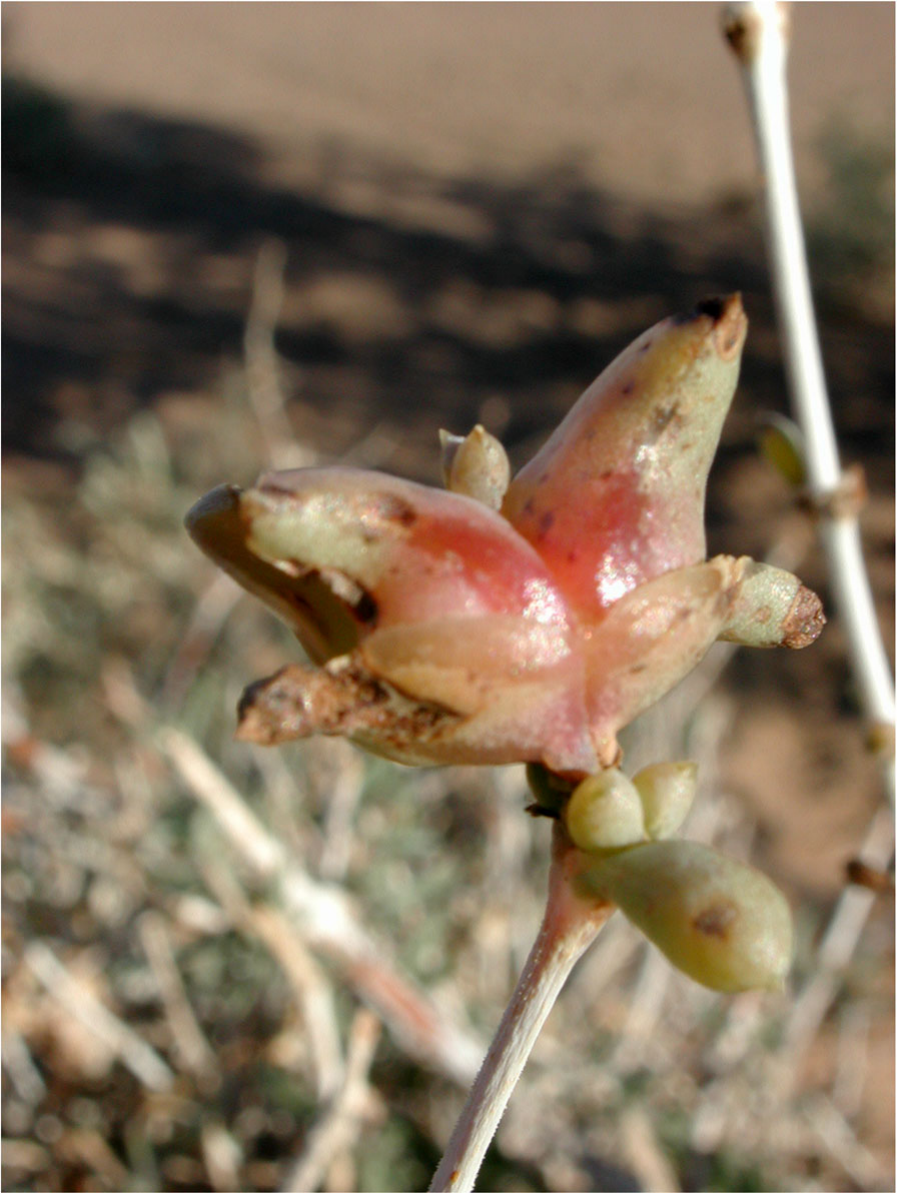
Fruit of *N. perrinii* (GV)

A brief botanical description of the plant is given by Ozenda [27] in his treatise on Saharan flora: the species is a shrub about 50 cm tall, characterised by opposed branches and leaves, fleshy and coriaceos leaves (Fig. 4), and small yellow flowers appearing axillary to leaves; stems are white with pinkish extremities, and a characteristic bell-shaped hard shell develops around the fruits (Fig. 5). The plant is endemic to the central and western Sahara desert where it grows in rocky and gravel plains either in flat, hilly, or mountainous areas. Sometimes, in salty depressions, it forms almost monospecific populations. It was first identified in 1900 by Battandier after the collections of Perrin in Touat and Tidikelt (Central Sahara) [40, 41]. It is known to Arabic speaking pastoralists as *askaf* (or *âskâf*, *āskāf*) [42] and to Tamachek speaking Tuareg as *tassak* [43].

**Fig. 6:**
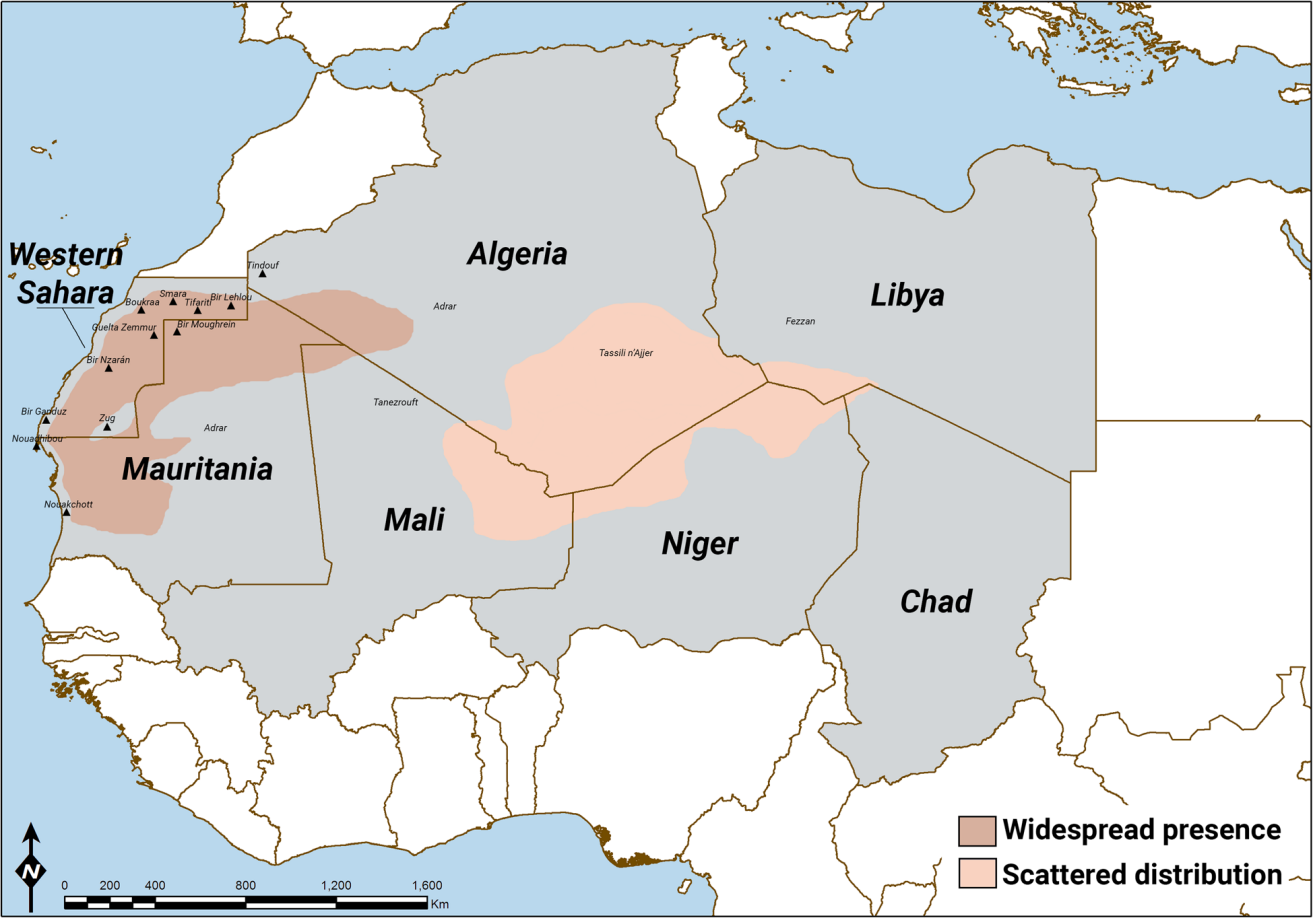
Map of the distribution of *N. perrinii*

Based on available data in botanical literature, ethnographic accounts, our observations during fieldwork, and of a map drawn by Quezel [44] (pg 514), we present in Fig. 6 a map of the distribution of *N. perrinii* within a geographical range that includes Libya, Chad, Niger, Mali, Algeria, Mauritania and Western Sahara. According to Lebrun [45], it is a western saharo-sindian species, along with other species such as *Crotalaria saharae* Coss., *Reseda villosa* Coss., and *Randonia Africana* Coss. These species live across western and part of central Sahara, and usually their range stops at east along a line between Bengasi and the Chad-Libya border [45]. *N. perrinii* ‘[does] not seem to spread into Libya towards the east’ [44] (pg 514). In fact, it is likely to reach its maximum diffusion in the rocky plains of western Sahara, where it is spread and abundant [46] and sometimes it forms monospecific populations, while in central Sahara it is described as present only ‘here and there’ in mountains and rocky plateaux (e.g. Tassili n’Ajjer) [47, 48]. It grows north until South Morocco [26, 27], disappearing when passing the northern limit of the Seguiet el Hamra (around the 27°00′ N parallel) [47]. Indeed, a study about camel feeding behaviour in Central-South Morocco (near Ouarzazate) did not report *N. perrinii* among local forages, where it is instead substituted in its role by species of the same family, e.g. *Salsola* and *Traganus* species [49]. The plant is present in a scattered way in the Bir Lehlou area, while it is completely absent from the Hamada of Tindouf. Adamou reports, in his paper about camel husbandry in Algeria [50], that *N. perrinii* has disappeared from the region of Tindouf due to the degradation of the nomadic circuits; however, we rather argue that if the plant has ever been present in the Tindouf region, this was only in its southern part, across the border with Mauritania and Mali. According to our observations in Western Sahara, its northern limit in this area is north of Bir Lehlou, at about the 26° 20′ N parallel. According to the Sahrawi herders, the above defined northern limit corresponds to a dry riverbed called *afrijed* not far from Bir Lehlou. Moving southward, populations of *N. perrinii* become abundant in the Zemmur areas of Bir Nzarán, Tifariti, Mehris, Guelta Zemmur, and Bir Moughrein (in the Mauritanian territory east of the Guelta Zemmour), especially in rocky plains, at the base of mountain ranges, and along the borders of dry riverbeds [25, 51]. In most of these areas, *N. perrinii* can be found commonly associated with *Panicum turgidum* in Acacia-Panicum steppes. Dobignard and colleagues [39] report *N. perrinii* in the hamada between Boukraa and Smara, in an ‘arid sandy steppe,’ and state that it grows south until the Mauritanian Adrar, becoming the dominant salt species in the southern part of the territory (Tiris) [11], where it grows in rocky steppes along with *Panicum turgidum* or *Stipagrostis* species. *Regs* (rocky deserts) with a predominance of *N. perrinii* are present east of Bir Ganduz (in the extreme south of Western Sahara territory) [46]. Across all its range, *N. perrinii* does not grow in predominantly sandy areas and where dunes appear (substituted by *Cornulaca monocantha* Delile); for example, it is reported as absent from the area south and east of Zug [46], where the Azéfal dune belt crosses the territory in direction north-east south-west. The western limit of its range are the oceanic coastal areas of Western Sahara and Mauritania, though it generally appears at some distance just of the coastal dune belts (the *aguerguer*, running in direction north–south just inland from the coast), and becomes progressively more abundant only towards the interior [25, 46, 52]. Outside Western Sahara, towards south and east, *N. perrinii* is present in discrete populations in non-dune areas of Central and North Mauritania, including the northern coastal area, where it is considered one of the best camel pastures [32, 53]. Monod [54] reports it between Aous and Jraif, in the French (Mauritanian) Adrar, just southeast from the border with Western Sahara, and it further seems to be an important species inland from Nouadhibou, while progressively becoming scarce moving further south. Naegelé [55] reports that *Arthrocnemum fruticosum* (L.) Moq. (Chenopodiaceae) is called ‘*askaf* of the South’ by Mauritanian nomads. Around Nouakchott, populations of *askaf* are reduced only to local *sebhas*, and the plant probably has its southern limit in the area around the 17° N parallel. The plant is also present throughout central and north Mali in sandy and rocky places, and again in areas of central Sahara as far east as the Libyan Fezzan and North Niger, as reported by Schulz [56] in his study of the flora and vegetation of that area. According to this author, *N. perrinii* is there the main representative (along with *Salsola baryosma* (Schult.) Dandy) of the Chenopodiaceae association, becoming rare towards north. *N. perrinii*’s presence in the area is reported between the 23° 56′ and 23° 15′ N parallels, and between the 11° 48′ and 10° 46′ E meridians [56]. The species is notably absent from the *tenefrut* (‘the desert of the desert’ separating Moorish/Sahrawi and Tuareg nomadic circuits, across the Algerian Adrar and part of Mali), from East Algeria (it is not reported among the pastures of Ghardaia and Ouargla, where it is functionally substituted by *C. monacantha* and *Traganum nudatum* Moq. [57]), as well as from central Niger (e.g. it is not reported among the camel pastures of Agadez) [58, 59]. In the areas of central Sahara, *N. perrinii* seems to be present with scattered populations, often limited to mountainous ranges [47]. This distribution hints at the possibility that the species was more widely distributed before the desertification of the Sahara began about 5,000 years ago, and that with increasing aridity its range restricted to (relatively wetter) mountainous ranges in central Sahara and toward the Atlantic coast in western Sahara.

### Ethnobotany and ethnoveterinary

*N. perrinii* is used by the Sahrawi for medicinal and ethnoveterinary purposes. For centuries the nomads have used plant and animal resources from the desert for medicinal purposes, and *N. perrinii* makes no exception. The Sahrawi smash the fresh leaves of *askaf* and mix them with water to form a poultice applied topically to treat skin infections and wounds [17]. The dried and powdered stems are added to tea as digestive and for stomach ache and abdominal pain, while the dried fruit is added to tea in a similar way to treat diabetes and hypertension. *Askaf* is also important as a firewood source, especially to prepare tea and as a fire-initiator [22]. In its ecological role in the western Sahara ecosystem, it is sought after by wild ungulates such as gazelles, which used to constitute an important food source to Sahrawi nomads. *N. perrinii* is also used as an ethnoveterinary remedy to treat sick camels. The Sahrawi regard *askaf* grazing to be a treatment for several camel diseases such as knee inflammations, *buguashish* and *zoran* (i.e. salt deficiencies), and *mindi* (i.e. intestinal parasites). Aerial parts are burnt in front of the animal to treat skin ulcers from camelpox and respiratory infections [28].

**Fig. 7:**
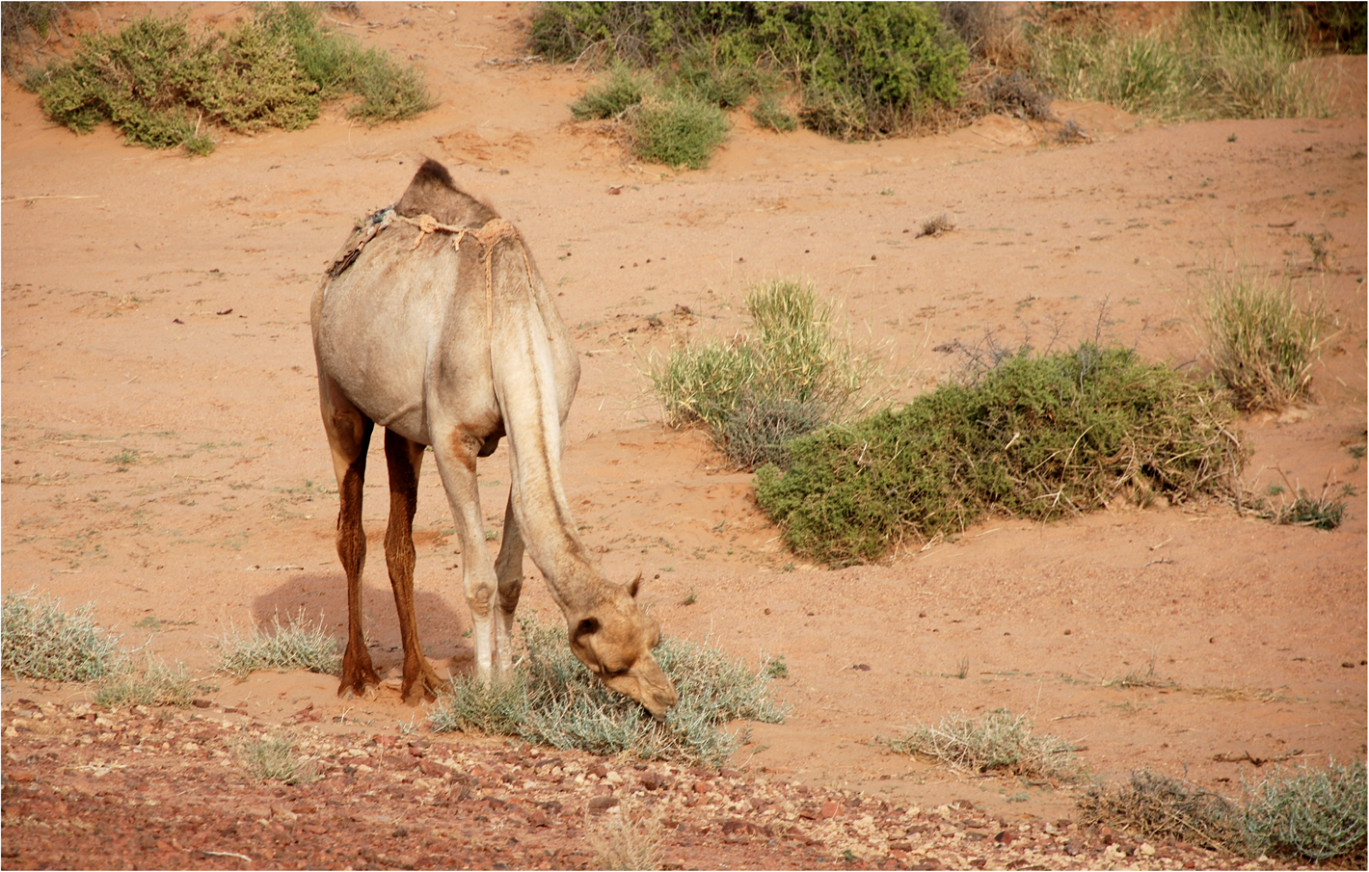
Camel grazing *askaf* (GV)

However, it is in its crucial role as camel pasture that *N. perrinii* displays all of its importance for the Sahrawi pastoral SES (Fig. 7). It is regarded as ‘the best and strongest pasture for camels,’ providing salts, strength and weight/fat to the animal. In their study of camel forages in Western Sahara, Volpato and Puri [21] found that *askaf* was the second most salient plant listed by Sahrawi herders [after *Acacia tortilis* and out of a total of 83 plants cited] and that it was mentioned by 75% of informants. Although *A. tortilis*, as well as *P. turgidum* and *Astragalus vogelii* Bornm. are also important plants for Sahrawi camel pastoralism, *N. perrinii* stands out as key forage due to its endemic distribution, to the crucial role as source of saline nutrients for western Saharan camels, and to being drought-resistant. Informants report that *askaf* is ‘available when no other forage does’ and associate its presence with camels’ health in terms such as ‘camels don’t fall sick if there is *askaf* to graze, and they recover from an illness if they graze *askaf*’, or again ‘with *askaf*, a camel can transport 400 kg from Tindouf to the Moroccan coast and back’ [21].

Camels graze often and preferentially on salty species, especially on plants with fleshy leaves that are high in protein and low in fiber content, and which develop green aerial parts during wet and dry periods alike [60, 61]. Due to camels’ need of six to eight times as much salt as other herbivores, halophytic plants represent the primary constituent of their diet, contributing up to one third of the total diet, with an even larger share during dry seasons and spells [32, 60]. Western Saharan camels get much of their salts from *N. perrinii*, which plays a fundamental role in camel diets, especially in the cold season, i.e. before annuals have sprouted [10, 62, 63]. During this period, nomads may lead their herds to a ‘salt cure’ based on *N. perrinii*, a 45-days diet that ‘cleans camels’ blood’ due to its purgative effect [23]. A similar importance is attributed to *askaf* by Moorish nomads of northern Mauritania, as reported by Correra [32] in his study of the dynamics of forage utilization in the Bank D’Arguin National Park. Local nomads report that *askaf* is one of the best pastures for camels, especially between January and October, corresponding to the local long dry season. Nomads and their camel herds from central Mauritania and Atar move north to Tiris and northern Mauritania once a year for salt-cures based on *N. perrinii*. According to one Sahrawi informant, ‘camels down there smell *askaf* from very far, and at the right period every year they want to move towards it.’ ‘Salt cures’ by means of rotational visits to specific salt-water wells, to salty soils, or to areas with association of halophyte plants is an husbandry practice pursued by several African nomadic pastoralists (e.g., Moors, Tuareg, and Somali) [61, 64, 65].

Thus, *N. perrinii* is not only one of the most important plants of the everyday grazing of camels in western Sahara, but it also defines specific periods during the nomadic year in which it predominates in camels’ diet and patterns of mobility are based on its distribution and availability. As reported by Boyer [23], Gauthier-Pilters [66], and Volpato and Puri [21], four nomadic seasons and respective patterns of camel dietary preferences can be distinguished in western Sahara; *askaf* is a main forage plants throughout the year, but it is during summers (from the beginning of June to the beginning of September), before the rains, when camels rely heavily on *N. perrinii*, that the plant stands out as a main element that beats the passing of time in Sahrawi nomadic lives.

### Ethnotaxonomy

Given the importance of *N. perrinii* for camel health and survival among the Sahrawi, it is not surprising that the plant is a main element of their plant ethnotaxonomy and classification. This is true both for camel forages’ classification as well as for general plant classification: *askaf* is the prototypical plant both for the category of *summa* plants (i.e. halophyte shrubs in generic classification) and of *hatba* or *m*’*hatba* plants (i.e. halophyte shrubs in camel forage classification) [21, 32]. *Hatba* includes thirteen species of salty forage plants (e.g. *Salsola* species, *Traganum nudatum*, *Cornulaca monacantha*) mostly belonging to the Chenopodiaceae family [21]. Distinct plants of *hatba* are preferentially distributed across different areas: *N. perrinii* grows in the rocky steppes of the interior, *C. monacantha* grows in sandy soils and dune areas, and *Atriplex halimus* L. grows along the coast [39].

The Sahrawi distinguish a ‘true askaf’ (simply called *askaf*) from a ‘false askaf’ (*askaf el haibe*). ‘True askaf’ is said to grow only in Western Sahara and north-central Mauritania, whereas ‘false askaf’ is allegedly weaker (in terms of power as forage, e.g. nutritional properties) and smaller, and grows in south Algeria, northern Mali, and other parts of central Sahara (i.e. out of the Sahrawi’s customary nomadic territory). The Sahrawi explain these differences on the basis that, in those surrounding areas, *askaf* has been historically disseminated through camel dung, and ‘not because it belongs there.’ False *askaf*’s weakness and smaller size are then attributed to the different way of propagation of the species: dung versus seed. According to one informant, ‘In Algeria there may be some *askaf*, but it is not as good as the one here [in Western Sahara], the plant in Algeria is weak, small, and is brought there by livestock. *Askaf* grows perfect only in Sahrawi territory.’ Partly, this distinction supports and is supported by phytogeographical information presented above, and it is shown in Fig. 6 in terms of distinct color tones: *N. perrinii* is distributed in a main areal of western Sahara and a secondary areal of central Sahara; in the former, the species displays higher biomass production (i.e. it grows more) and higher abundance (i.e. individual plants per area) and hence it is key to local camel husbandry, while in the latter *askaf* is smaller and scattered and functionally substituted in its role by other halophyte species. Not by chance, the primary area of distribution of *N. perrinii* corresponds quite neatly with the customary grazing territory of the Sahrawi tribes, and the tribes before, since about 1,500 years. Thousand-year-old camel nomads of western Sahara, much like Sahrawi refugees today, struggled to get access to this important forage plant with their camel herds.

### Askaf and camel diseases

The Sahrawi recognize strict and complex relationships between their camel herds and the desert ecosystem in terms of health [67]. They aim at rationally coupling the needs of their camels with the ever changing variability of grazing resources in time and space. Much of this rationale is related to and uses *N. perrinii* as term of reference and symbolic representation of the good characteristics of Western Sahara for camel husbandry versus the poor characteristics of surrounding areas. *Askaf* is central to the concept of health and disease as its distribution define territories where certain diseases are present or absent. In this way, *askaf* and camel diseases become also representative of a customary nomadic territory. Boundaries are defined conceptually and physically (i.e. by the presence of *N. perrinii*), and on these boundary-forming elements Sahrawi cultural identity is built and understood by nomads and refugees alike.

Among the diseases conceptually related to *N. perrinii*, the most important is *buguashish*, a salt and mineral deficit manifesting in lameness, progressive weakening, and bone fragility that results in spontaneous fractures, and caused by pasturing or traveling in sandy areas of Central Mauritania, Southwest Algeria, and Mali [28, 68]. Informants conceptualize *buguashish* in terms of presence or absence of *N. perrinii*. Given the importance and geographical distribution of *N. perrinii* in Western Sahara, Sahrawi herders tend to associate its presence with healthy camels and its absence (e.g. to the north, east, and south) with mineral deficits and disease. Therapy relies in moving camels to areas where they can graze *askaf* (or to the rocky soils where the species grows). Informants state that ‘*buguashish* is a problem of the camels of the East, which do not eat *askaf*; is a problem of camels from sandy soils and dunes, where *askaf* does not grow; it occurs to camels that spend one or more years without eating *askaf*.’ Mineral deficits (e.g. NaCl deficiency, phosphate/calcium disequilibrium) and associated health conditions (e.g. kraff disease, pica-pica) are relevant in many desert areas where camels live [65, 69–72]. These deficits especially occur when camels graze for long periods in phosphorus-poor soils. Camel health is instead supported by grazing in halophyte-rich areas, i.e. where *N. perrinii* is abundant. Thus, the conceptualization of *buguashish* in Sahrawi culture, in absence of knowledge of the phosphorus and its role, is built around a counterposition of positives (*N. perrinii* and other salty plants, healthy status, rocky soils) and negatives (no *N. perrinii*, fractures and lameness in camels, sandy soils, e.g. the dune areas at the eastern periphery of their customary nomadic territories) [28].

Other conditions associated with the presence/absence of *N. perrinii* include *homzi* and *ghesh* (parasites of the respiratory system) to the north, and trypanosomiasis to the south and north [28]. Although it is one of the diseases most dreaded by African camel pastoralists [73, 74], trypanosomiasis is notably rather absent from the Sahrawi customary territories. It is instead generally present during rainy seasons in most of the remaining camel husbandry areas including Morocco (Tafilalet and Ouarzazate provinces), Central and Southern Mauritania, Mali, Chad, and Niger [75–77]. It appears that seroprevalence progressively decreases with decreasing annual precipitations towards the inner Sahara desert, whilst it is probably negligible in drier areas of Western and Central Sahara [28]. Sahrawi customary grazing areas are historically reported as free from trypanosomiasis [23, 68]. According to informants, death of trypanosomiasis-affected camels can be delayed or health restored if they are moved to graze from *N. perrinii*, which is here used as exemplificative of the trypanosomiasis-free Western Sahara ecosystem (i.e. if camels graze from *N. perrinii* it means that they are in Western Sahara and thus not exposed to trypanosomiasis vectors). Cures of salty plants are reported as treatments of trypanosomiasis by Monteil [68] and Curasson [78]. Like for *buguashish*, the Sahrawi’s understanding of trypanosomiasis is embodied in the contrast between their customary areas (where the disease is absent and *N. perrinii* is abundant) and areas further to either the South or the Northwest Sahara (where the disease is present and *N. perrinii* is absent). Sahrawi mental map in relation to camel pastures is strictly related with the mental map associated to camel illnesses: ‘Camels of Western Sahara are stronger and healthier; those coming from the East [e.g. Mali, Niger] are full of *mahuar* [scars from cauterizations for veterinary purposes]’. In essence, ‘*Askaf* is a pasture that treats camel diseases; a camel can live thirty years here without getting sick as long as there is *askaf* and some rain.’

### Askaf and the taste of camel milk

The importance of *N. perrinii* for the Sahrawi is reflected in the organoleptic characteristics it gives to camel milk, Sahrawi’s staple food. The Sahrawi recognize in detail the relations between forages and the taste, smell, or health and nutritional properties of camel milk. Sahrawi practices around the taste and smell of camel products are embedded in Sahrawi cultural values and identity [21].

The taste and smell of the plants grazed in each area and season is reflected in the taste and smell of milk. Taste and smell are representative of, and attached to, customary grazing areas, and hence also an element of cultural identity. *N. perrinii* is exemplary in this process of cultural identity construction, and Sahrawi nomads perceive that the ‘perfect’ light-salty taste of *N. perrinii* in camel milk is characteristic of Western Sahara, in contrast with other nomads (e.g. Tuareg), their territory (where the plant is absent), and the taste of that milk. Consuming milk from an animal that grazed from *N. perrinii* is regarded as pleasant due to its good taste, and healthy due to the medicinal and nutraceutical properties of the plants ‘embedded’ into the properties of the milk.

**Fig. 8:**
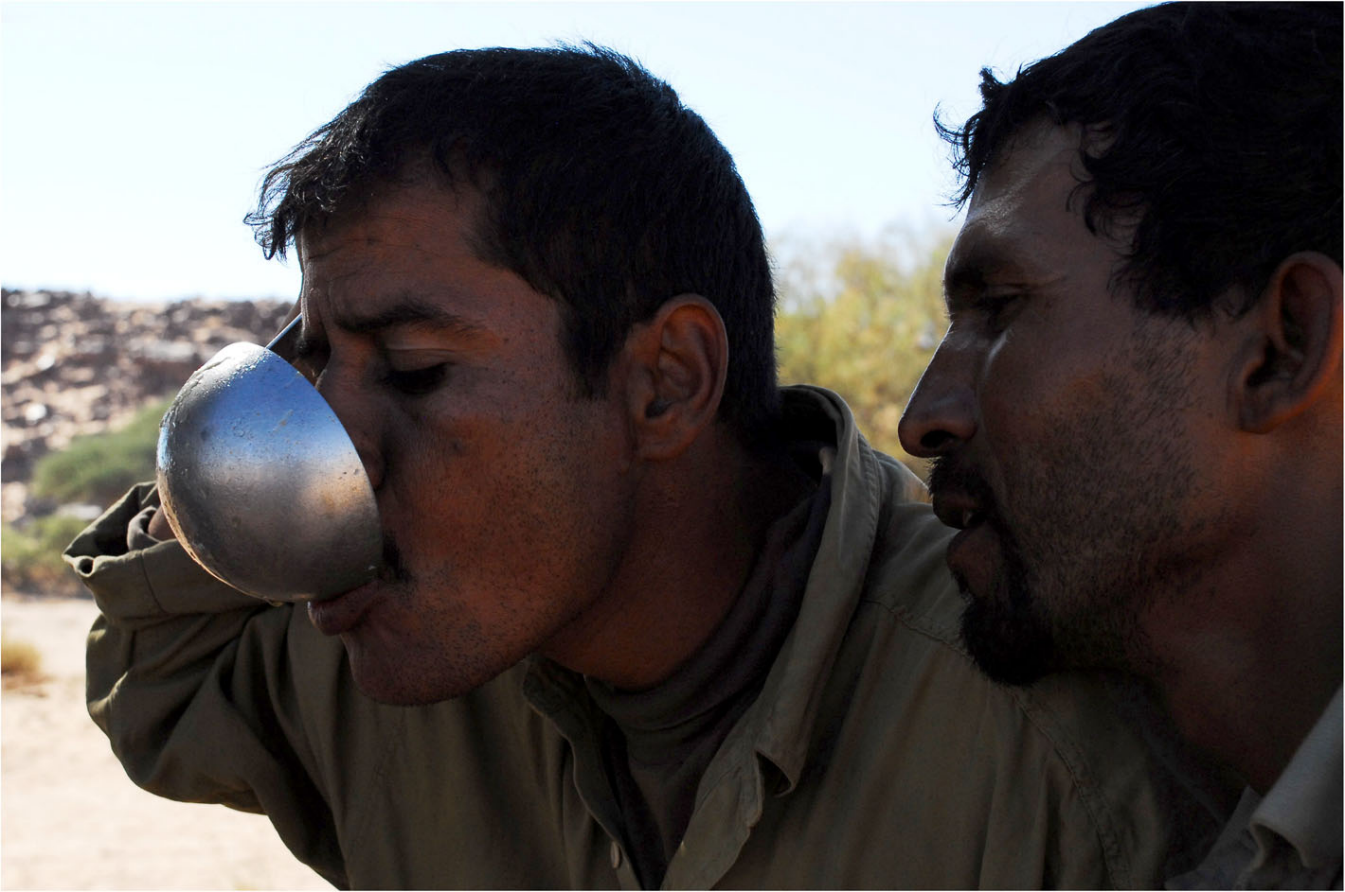
Refugees drinking camel milk (GV)

**Fig. 9:**
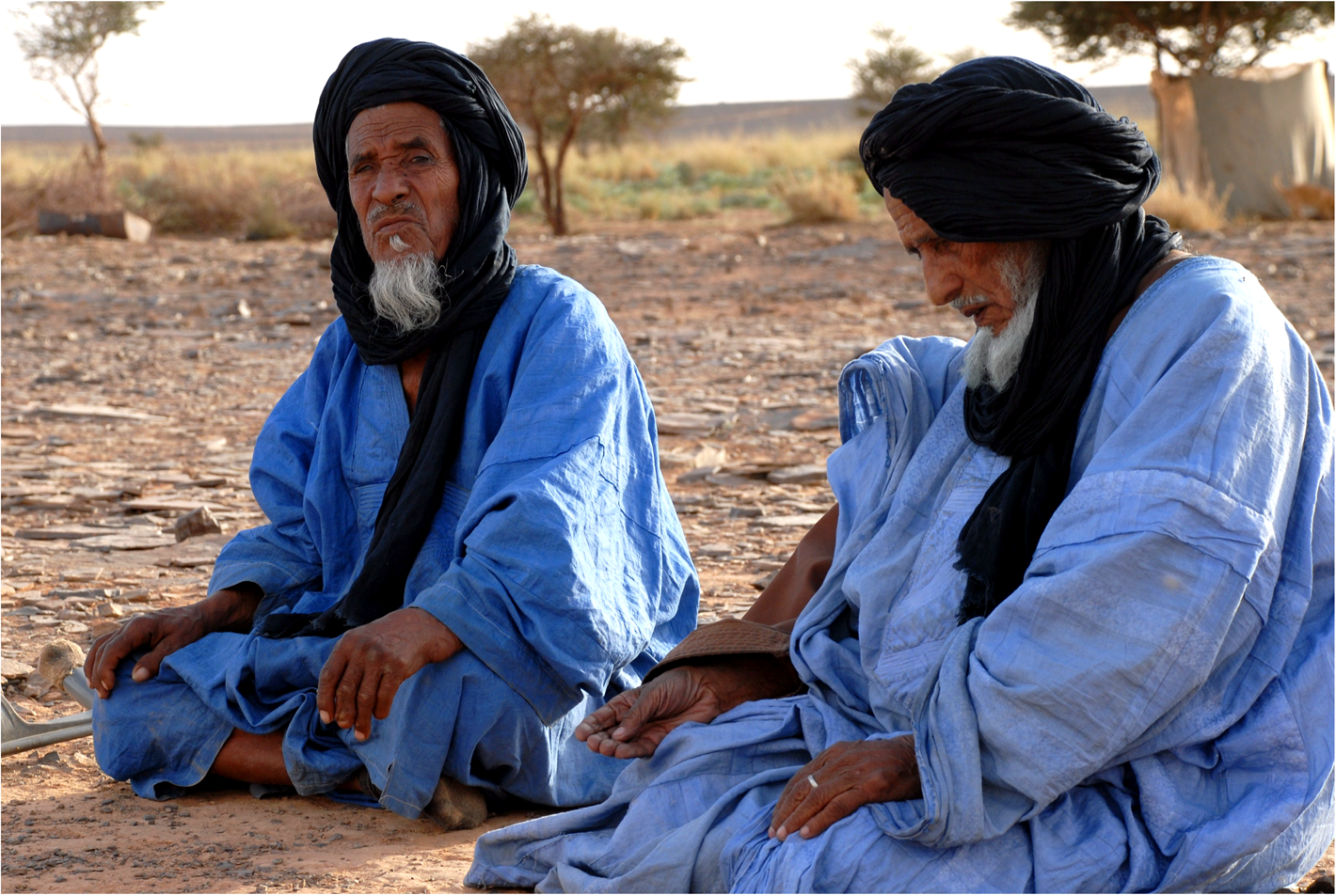
Old refugees in the former nomadic territories (GV)

In these tastes and smells, the Sahrawi nomads and refugees recognise those of their (lost and partly recovered) homeland, and further associate them with concepts such as freedom (e.g. of crossing the desert with camels), belonging (e.g. to a group of people with specific food practices), and dignity (i.e. an individual or groups sense of self-respect and self-worth). Culturally-relevant foods and their taste are sought beyond their nutritional and health properties, and food habits, preferences, and taste are constituents of values and cultural identities. Refugees pursue productive and market activities and use subsistence resources to reduce dependence on food aid and obtain ‘their’ foods. In this way the taste of these foods is related to the struggle of the Sahrawi refugees to recover their culture and livelihoods. Furthermore, the Sahrawi distinguish four nomadic seasons and respective patterns of camel dietary preferences. In each season, different main grazing plants give different taste to the milk. The taste of the milk becomes representative of the rhythms of seasons and years dictated by camels and local desert ecologies, and not by camps’ limbo and international policies, or by queues to get food aid. Thus, milk taste represents time, space, and associated values and feelings. Refugees contrast fresh camel milk and its taste with powder milk. The first represents ‘life as it should be,’ it is socially accepted and culturally meaningful (Fig. 8). The second, received as food aid in the camps, represents powerlessness, alienation, and loss. Fresh camel milk makes life in the camps less distressing, re-activating hope and sense of belonging. One informant emphatically explained: ‘The best life is when you are with camels in an area with *askaf* and you drink the milk from those camels’. Drinking milk with the distinctive taste given by *askaf* and/or other pastures of the nomadic territories is also a main desire of elderly in the camps. Bringing one’s elderly to the customary nomadic areas to drink fresh camel milk is then a mayor social expectation that refugees try to meet by acquiring camels and through temporary nomadism. This provides older refugees with a much-desired glimpse of their pre-war nomadic life and its products (Fig. 9).

### Askaf and cultural and political identity

Sahrawi cultural identity is related to the central elements of their nomadic livelihoods and to the territory where these take place. *N. perrinii* is a main element of this identity. Among the elements that symbolically define their own pastoral SES, the Sahrawi include *askaf*, as well as camel-based subsistence products and camel-associated skills and knowledge [16], and specific camel breeds [79]. *Askaf* becomes a claim to a national territory delimited by its presence, where extensive camel husbandry and associated knowledge, culture, and values are pursued since hundreds of years.

Nomadic tribes substantially engage with each other in order to have access to grazing areas (i.e. with better grazing resources, absence of diseases and vectors, etc.). Resources and characteristic features of these areas are foundational to tribes’ cultural identity (and capacity of collective mobilization to defend those grazing areas). This is the case with *N. perrinii* and its centrality in ‘camel narratives’ among the Sahrawi. The occupation of areas fit for camel husbandry is conceptualised around the absence of certain diseases (e.g. trypanosomiasis, *buguashish*), and understood in terms of contraposition between areas where these diseases occur and their characteristics (e.g. sandy areas, wetter areas, areas without *askaf*) and customary grazing areas (Western Sahara and northern Mauritania, presence of *askaf*). The set of relations established in the *badyia* are contrasted with the predominant relations in the outer (strangers’) territories, and embedded into Sahrawi cultural roots. So, the ‘ideal picture’ of Sahrawi camel husbandry is characterized by absences as well as presences: *N. perrinii* and its taste in camel milk are defining presences; trypanosomiasis, *buguashish* and other diseases are defining absences.

The Sahrawi construe a further contraposition in terms of differences between the refugee camps and the customary grazing territories of Western Sahara, in terms of presence/absence of *askaf*, constraints in camel husbandry, and taste of camel milk. These contrapositions are embedded into a narrative that symbolically represents refugee camps as places where traditional livelihoods can’t be pursued, where camels do not live and produce at their best, where camel products do not have the ‘right taste’, and where the food obtained through aids (e.g. powder milk) do not adhere to Sahrawi gastronomic standards.

In surrounding nomadic areas, there are regions characterised by the presence of specific threatening camel illnesses, unfavourable environmental conditions, and/or unfavourable political situations. Among these territories, we note South Morocco, in the regions North of Seguia el Hamra and of Tarfaya. There, it historically existed a geographical border between different SESs, with SESs based on sedentary small livestock breeding and small-scale farming to the north, and nomadic camel pastoralism to the south, through a continuum of diversified smaller-scale SESs. In these areas of Southern Morocco, the Sahrawi state that the risk for camels of getting illnesses such as *buguashish* or *homzi* increases, important camel pastures are absent, and general environmental conditions are less favourable for camel breeding (i.e. higher humidity and oceanic climate). Given the context in which Sahrawi nomads became refugees and the conditions of life in the refugee camps, these constructions around *N. perrinii* become a statement of political identity: through the narratives around *askaf*, the Sahrawi politically claim their customary territory as homeland.

## Conclusions

Pastoral social-ecological systems worldwide are rooted in the livestock species and in the grazing resources available in a territory and utilized through the deployment of different forms of mobility within a system of social relations and culture. This study has provided an account of a key species and its relations using as case study the role and importance of the grazing plant *Nucularia perrinii* for the camel-based pastoral systems of the Sahrawi nomads and refugees of Western Sahara.

We reported first on the botany of *N. perrinii* in terms of Saharan distribution and on its taxonomy among Saharan pastoralists, and then on its material (i.e. as camel forage and veterinary remedy) and cultural (i.e. to define customary grazing territories, as main element of cultural identity) importance among the Sahrawi. This halophyte species, called *askaf*, is understood by the Sahrawi as representative and emblematic of the goodness of Western Sahara environment for camel husbandry. Its populations define the boundaries of customary grazing territories, and as such narratives about *askaf* are embedded into conceptualisation of camel diseases and their distribution, into Sahrawi gastronomic and cultural standards (i.e. in the taste given by *askaf* to camel milk), and further into Sahrawi identity. The counterposition that the Sahrawi construe between *askaf* presence, on the one hand, and with its absence and the presence of camel diseases on the other hand signals that *askaf* is embedded into Sahrawi cultural identity. The counterposition of *askaf*-tasting camel milk with powder milk made available in the refugee camps by food aid signals the antithesis between the positive values of nomadic life and the negative values of life as refugees in the camps. As a whole, these results suggest that *N. perrinii* is used by the Sahrawi as important grazing resource but also as a tool in which political narratives of territorial claims and self-determination (versus Morocco for the Western Sahara, and versus the refugee camps for nomadic livelihoods based on freedom of movement) are woven.

We conclude that *N. perrinii* ties the ecology of the Western Sahara desert with camel husbandry and associated livelihoods, and further with the culture and worldview of the Sahrawi nomads and refugees. We contribute to the understanding of the key roles of grazing species in extensive pastoral SESs, and of these species’ importance for subsistence livelihoods. These findings enhance our understanding of the critical role that specific pastures have in pastoral SESs, which not only provide sustenance to pastoralists’ herds but are also cultural elements woven into the fabric of pastoralists’ values, beliefs, and identity.

## Footnotes

1 Throughout this paper, we use the definition ‘western Sahara’ when referring to the western part of the Sahara desert, while we spell ‘Western Sahara’ when referring to the country (former Spanish Sahara) and its territory. Western Sahara is thus a politically-delimited part of western Sahara.
